# Identification of *Escherichia coli* 166 isolate as an effective inhibitor of African swine fever virus replication

**DOI:** 10.1128/spectrum.03009-24

**Published:** 2025-02-26

**Authors:** Jinya Zhang, Hongyu Cui, Zhenjiang Zhang, Wenqing Wang, Fengwei Jiang, Encheng Sun, Yuanmao Zhu, Fang Li, Zhigao Bu, Dongming Zhao

**Affiliations:** 1 State Key Laboratory for Animal Disease Prevention and Control, National High Containment Facilities for Animal Diseases Control and Prevention, National African Swine Fever Para-reference Laboratory, Harbin Veterinary Research Institute, Chinese Academy of Agricultural Sciences, Harbin, China; Michigan State University, East Lansing, Michigan, USA

**Keywords:** African swine fever virus, *Escherichia coli *166, wild boar, antiviral, NF-κB, CD163

## Abstract

African swine fever is a lethal disease with mortality rates approaching 100% in both domestic pigs and wild boars. With no effective vaccines or treatments available, there is an urgent need for new biologics to combat the African swine fever virus (ASFV). In this study, we isolated bacteria from the intestinal contents of wild boar using culture-based methods and identified them through 16S ribosomal DNA (rDNA) sequencing. These isolates underwent high-throughput screening to evaluate their immunomodulatory effects on J774-Dual cells and their ability to inhibit ASFV replication *in vitro*. Among them, an *Escherichia coli* strain, designated as *E. coli* 166, exhibited strong inhibitory effects on various ASFV strains’ replication, including three genotype II strains: virulent strain HLJ/18, moderately virulent strain HLJ/HRB1/20, genetically modified low-virulent strain HLJ/18–6GD, and one genotype I low-virulent strain SD/DY-I/21. Notably, this inhibition did not require direct interaction between the bacteria and porcine alveolar macrophages (PAMs). Both live and heat-inactivated *E. coli* 166 demonstrated a strong inhibitory effect on ASFV replication. Genetic modification of *E. coli* 166 did not alter its inhibitory phenotype. Further analysis revealed that PAMs pretreated with *E. coli* 166 showed upregulation of NF-κB and downregulation of CD163 at different time points post-infection, whereas PAMs only infected with ASFV exhibited the opposite trend. These findings suggest that *E. coli* 166 holds promise as a biological agent for controlling ASFV infection, through indirect mechanisms involving bacterial metabolites or lysis products. Future studies should focus on identifying the specific components responsible for its antiviral effects.

**IMPORTANCE:**

The emergence of the African swine fever virus (ASFV) as a devastating pathogen in swine populations necessitates the development of novel strategies for its control. In this study, *Escherichia coli* strain 166 (*E. coli* 166) demonstrated a remarkable ability to inhibit the replication of multiple ASFV strains in porcine alveolar macrophages (PAMs), even without direct bacterial contact. Both live and heat-inactivated *E. coli* 166 retained this inhibitory effect, suggesting that secreted metabolites or lysis products may play a key role. Furthermore, pretreatment of PAMs with *E. coli* 166 resulted in upregulated NF-κB activity and downregulated expression of the ASFV entry receptor CD163, presenting an immune-modulatory mechanism distinct from PAMs solely infected with ASFV. These findings highlight the potential of *E. coli* 166 as a biological agent to combat ASFV, offering a promising alternative or complementary approach to traditional antiviral strategies.

## INTRODUCTION

African swine fever (ASF) is a devastating disease caused by the African swine fever virus (ASFV) that affects both domestic pigs and wild boars. ASF is a notifiable disease to the World Organization for Animal Health (WOAH) (1) and is currently regarded as the most serious constraint on pig production. Initially identified in Kenya in 1921, ASF has spread to various regions, affecting over 80 countries across Africa, Asia, Europe, and more recently, Oceania (2). The ASFV genome is classified based on the sequence variation in the C-terminal region of the *B646L* gene, which encodes the p72 protein. To date, at least 24 distinct genotypes have been identified worldwide.

The first ASF report in China was occurred in 2018. Since then, the epidemiological situation has varied significantly. Surveillance studies have identified several strains, including genotype II highly virulent Georgia07-like strains, genotype II moderately virulent natural mutants, genotype I low-virulent strains, and recombinant strains of genotypes I and II, complicating early detection and control efforts for ASF (3–6). The live attenuated vaccine candidate derived from genotype II ASFV (HLJ/18–7GD) does not provide protection against the challenge of genotype I and II recombinant viruses (3–5, 7). Current control measures for ASF primarily rely on following strict biosafety measures and culling infected animals, underscoring the urgent need for effective vaccines or antiviral treatments. This situation highlights the urgent need for alternative strategies to combat ASFV.

Research has demonstrated that gut microbiota significantly modulates host resistance to ASFV. Fecal microbiota transplantation from warthogs can impact pigs’ susceptibility to ASFV, providing partial protection against attenuated ASFV (8). Additionally, *Bacillus subtilis* has been shown to partially inhibit ASFV infection both *in vivo* and *in vitro*; its metabolites, arctiin and genistein, interfere with the function of viral topoisomerase II (9). Furthermore, Food and Drug Administration (FDA) approved drugs like triapine and cytarabine hydrochloride have been found to synergistically inhibit ASFV replication (10). Natural compounds, such as kaempferol, inhibit ASFV replication at non-cytotoxic concentrations, potentially affecting multiple stages of the viral lifecycle, tested in Vero cells and porcine macrophages (11). Apigenin and genkwanin inhibit ASFV by disrupting viral DNA synthesis and entry processes, tested in Vero cells (12, 13). Tetrandrine inhibits ASFV by blocking macropinocytosis during the internalization phase of the virus (14). The discovery of natural biologics that can inhibit ASFV replication presents a promising avenue for disease control.

In this study, we investigated the potential of *E. coli* 166, an isolate from wild boar intestinal contents, as a biological agent against ASFV. Through a series of *in vitro* experiments, we identified *E. coli* 166 as a potent inhibitor of ASFV replication. This inhibition was observed across multiple ASFV strains, suggesting that *E. coli* 166 may serve as a viable candidate for biological control of ASFV. Further analysis with a transwell coculture system revealed that the inhibitory effect of *E. coli* 166 did not require direct interaction with porcine alveolar macrophages (PAMs), and heat-inactivated *E. coli* 166 is with same inhibition effect as alive *E. coli* 166, indicating that the released compounds could play a crucial role in this process. As *E. coli* 166 is isolated from wild boar intestinal contents, it presents fewer challenges related to colonization compared to isolates from other breeds.

## RESULTS

### High-throughput screening of the immunoregulatory effects of wild boar bacterial isolates on J774-Dual reporter cells

**Fig 1:**
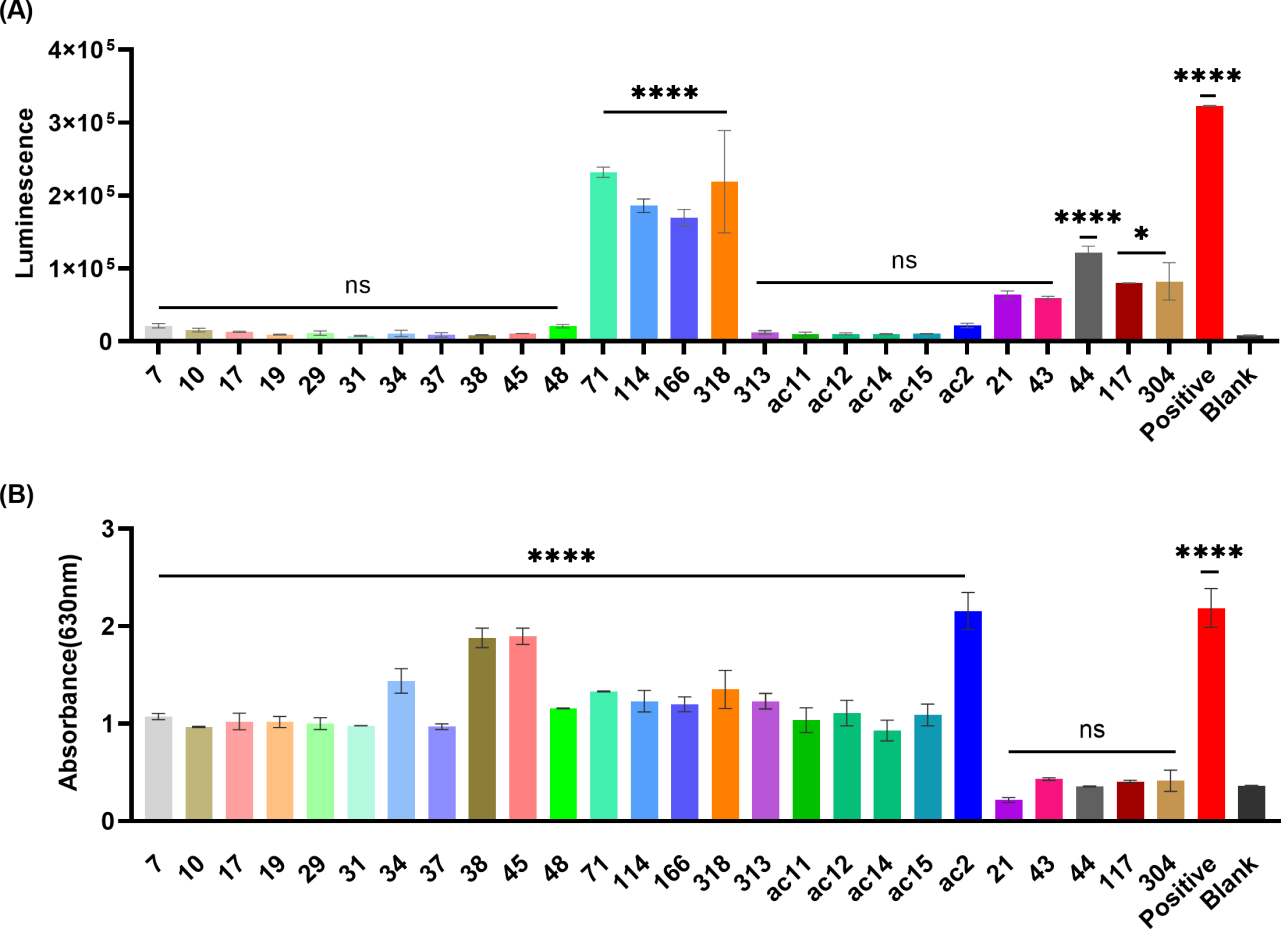
Characterization of immunoregulatory effect of wild boar bacterial isolates on J774-Dual reporter cells. Analysis of (**A**) IRF and (**B**) NF-κB signaling pathway activation in live bacterial isolates interacting directly with J774-Dual cells for 24 h. Each number on the X axis represents one isolate. Bacteria:cell ratio is 50. Statistical significance was defined as *P < 0.05, **P < 0.01, ***P < 0.001, and ****P < 0.0001. ns, no significance.

J774-Dual cells allow for simultaneous study of the NF-κB and interferon regulatory factor (IRF) signaling pathways (15). To assess immunostimulatory effect of wild boar bacterial isolates, high-throughput screening was conducted with live bacteria through direct interaction with J774-Dual cells. The IRF signaling pathway was significantly activated upon stimulation with isolates 71, 114, 166, 318, and 44 compared to the blank control, while a less pronounced response observed for isolates 117 and 304. The NF-κB signaling pathway was significantly activated by isolates 7, 10, 17, 19, 29, 31, 34, 37, 38, 45, 48, 71, 114, 166, 318, 313, ac11, ac12, ac14, ac15, and ac2 compared to the blank control. Notably, four isolates 71, 114, 166, and 318 were capable of activating both the IRF and NF-κB signaling pathways strongly (Fig. 1). These four isolates were selected for further analysis.

### Enterobacterial repetitive intergenic consensus PCR analysis of selected isolates

**Fig 2:**
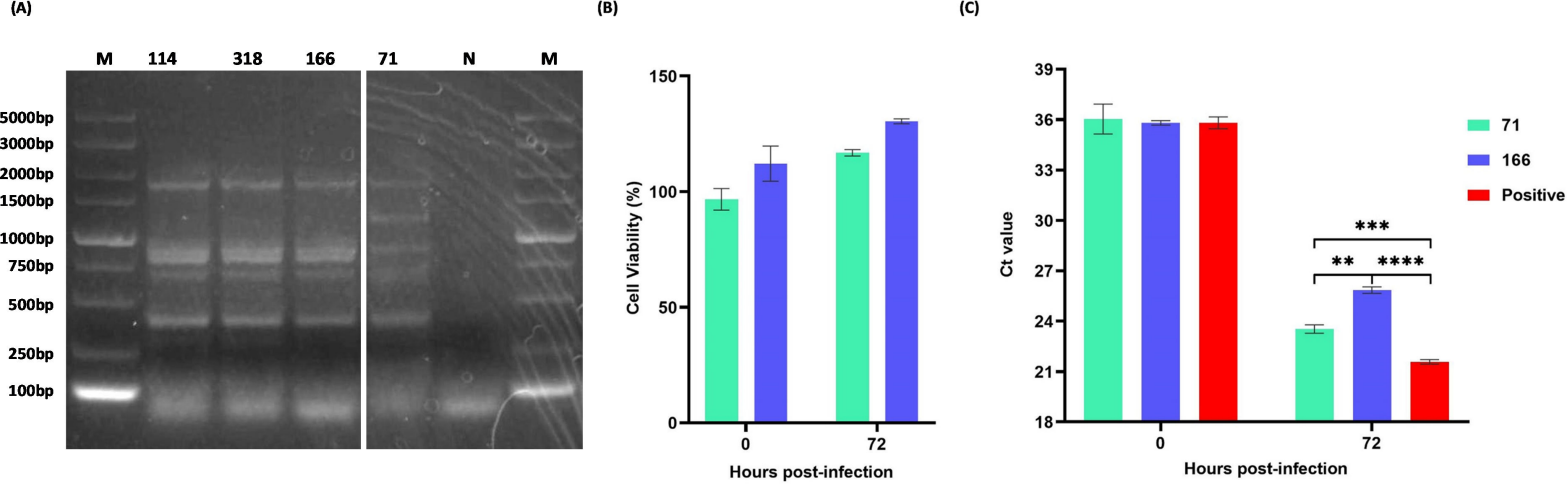
ERIC-PCR checking of isolates 114, 318, 166, and 71, cytotoxicity effect and inhibitory effect on the replication of HLJ/18–6GD of isolate 71 and 166 on PAMs. (**A**) Gel electrophoresis analysis of ERIC-PCR fingerprinting of isolates 114, 318, 166, and 71. Lane 1 was marker, lane 2 was 114, lane 3 was 318, lane 4 was 166, lane 5 was 71, lane 6 was negative control, and lane 7 was marker. (**B**) Cytotoxicity analysis of isolate 71 and 166 interacting directly with PAMs at 0 and 72 hours post-infection (hpi). Bacteria:cell ratio is 50. The threshold of cell viability is 90%, cell viability less than this is considered as toxic, and higher than this is considered as not toxic. (**C**) Comparison of inhibitory effect on the replication of HLJ/18–6GD of isolate 71 and 166 at 0 and 72 hpi. Statistical significance was defined as *P < 0.05, **P < 0.01, ***P < 0.001, and ****P < 0.0001. ns, no significance.

Based on 16S rDNA sequence results (Table S1), four isolates 114, 318, 71, and 166 were all blasted as *Escherichia* or *Shigella*. Gel electrophoresis analysis of enterobacterial repetitive intergenic consensus PCR (ERIC-PCR) showed that isolates 114, 318, and 166 exhibited similar fingerprints, indicating these three isolates were the same strain. These three isolates were different from isolate 71 (Fig. 2A). *E. coli* isolates 166 and 71 were selected for further analysis.

### Inhibition effect of *E. coli* isolates 71 and 166 on the replication of ASFV on PAMs

PAMs are widely used for *in vitro* ASFV studies. To investigate the cytotoxicity effects of *E. coli* isolates 71 and 166, the Cell Counting Kit-8 (CCK-8) assay was conducted through direct interaction of these bacteria isolates and PAMs. Both *E. coli* isolates 166 and 71 exhibited beneficial effects on PAMs at 0 and 72 hours post-infection (hpi) with a multiplicity of infection (MOI) of 50 (Fig. 2B).

We then evaluated the potential inhibitory effect of *E. coli* isolates 166 and 71 on ASFV replication and found that the replication of ASFV-enhanced green fluorescent protein (eGFP) was significantly suppressed by *E. coli* 166 (*P* < 0.0001) compared with that of positive control at 72 hpi, while *E. coli* 71 showed less inhibitory effect (*P* < 0.001; Fig. 2C). The inhibition effect of *E. coli* 166 is significantly higher than *E. coli* 71 (*P* < 0.01). Thus, further experiments were focused exclusively on *E. coli* 166 due to its notable antiviral properties.

### Inhibition effect of *E. coli* 166 on the replication of different ASFV strains on PAMs

**Fig 3:**
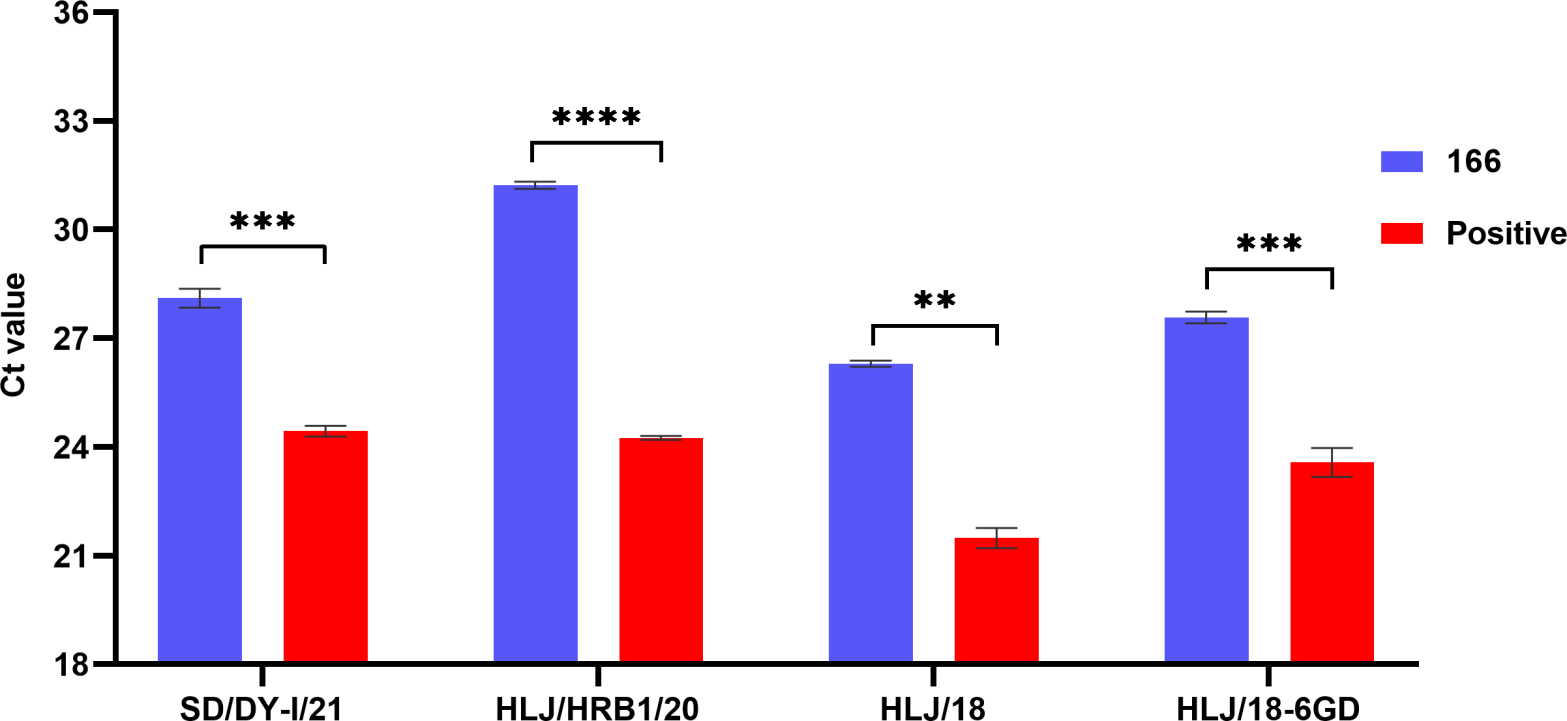
Inhibition effect of *E. coli* 166 on the replication of different ASFV isolates on PAMs. *E. coli* 166 significantly inhibited the replication of HLJ/HRB1/20 (*P* < 0.0001), of SD/DY-I/21 (*P* < 0.001) and HLJ/18–6GD (*P* < 0.001), and also inhibited the replication HLJ/18 (*P* < 0.01) when compared with PAMs only infected with ASFV. Statistical significance was defined as *P < 0.05, **P < 0.01, ***P < 0.001, and ****P < 0.0001. ns, no significance.

ASFVs with different virulence have been isolated from field in China (3–5). Given the significant inhibitory effect of *E. coli* 166 on ASFV-eGFP replication, we further extended our investigation to evaluate whether *E. coli* 166 affects the replication of other different ASFV strains or not. The viral genome in the supernatants was quantified using qPCR and compared. We found that *E. coli* 166 exhibited an inhibitory effect on all four ASFV strains tested. Specifically, *E. coli* 166 significantly inhibited the replication of the genotype I low-virulent strain SD/DY-I/21 (*P* < 0.001), the genotype II naturally mutated moderately virulent strain HLJ/HRB1/20 (*P* < 0.0001), the genotype II virulent strain HLJ/18 (*P* < 0.01), and the genetically modified low-virulent strain HLJ/18–6GD (*P* < 0.001; Fig. 3). These results confirmed the broad inhibitory potential of isolate 166 across various ASFV strains.

### Inhibition effect of different forms of *E. coli* 166 on ASFV replication on PAMs

**Fig 4:**
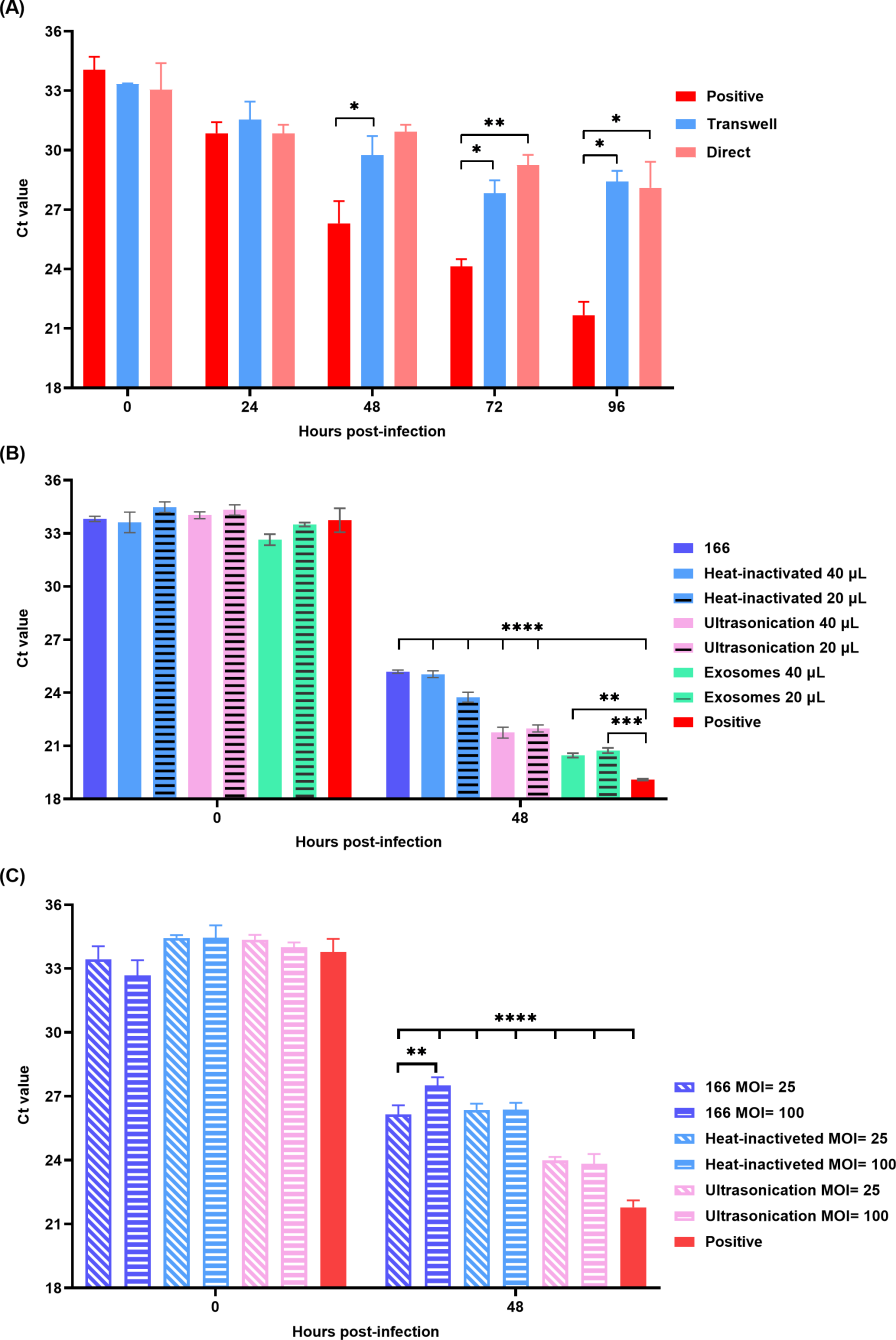
Evaluation of the inhibition effect of different forms of *E. coli* 166 on the replication of HLJ/18–6GD. (**A**) Comparative analysis of ASFV replication in the supernatant at 0, 24, 48, 72, and 96 hpi from ASFV-infected PAMs interacting directly (“direct”) or indirectly (“transwell”) with *E. coli* 166 to determine whether direct contact between the bacteria and PAMs is necessary for inhibition. (**B**) Alive *E. coli* 166 and heat-inactivated *E. coli* 166 showed the same inhibition effect, and there is no difference in the inhibition effect between alive *E. coli* 166 and heat-inactivated; ultrasonication product of *E. coli* 166 and exosomes of *E. coli* 166 could inhibit the replication of ASFV but less strong than alive and heat-inactivated *E. coli* 166. (**C**) Dose-response experiments using varying MOIs (25 to 100) and different preparations of bacterial lysates. The antiviral activity of live *E. coli 166* is dose-dependent. The bacterial lysates, generated through sonication and heat-inactivation of intact bacterial cultures, did not exhibit a dose-dependent antiviral activity. Statistical significance was defined as * *P* < 0.05, ** *P* < 0.01, *** *P* < 0.001, and **** *P* < 0.0001. ns, no significance.

When PAMs were treated with live *E. coli* 166, ASFV replication was effectively inhibited. To determine whether this inhibition required direct interaction between live *E. coli* 166 and PAMs, we utilized transwell which is a system commonly applied for assessing various host-pathogen and drug interactions (16–20). The results showed that, compared to the positive control (PAMs only infected with ASFV), ASFV replication was significantly reduced in the “direct” group (where *E. coli* 166 directly interacted with PAMs) at 48, 72, and 96 hpi (*P, **P, and *P, respectively). Interestingly, a similar trend was observed in the “transwell” group (where *E. coli* 166 indirectly interacted with PAMs) at 72 and 96 hpi (*P and *P, respectively; Fig. 4A), indicating that direct contact was not necessary for the inhibitory effect. This finding suggests that secreted metabolites or lysis products of *E. coli* 166 likely play a key role in suppressing ASFV replication.

Further experiments with different forms of *E. coli* 166, including heat-inactivated *E. coli* 166, ultrasonicated *E. coli* 166, and *E. coli* 166-derived exosomes, revealed comparable inhibitory effects on HLJ/18–6GD replication. The results showed no difference in inhibition between live *E. coli* 166 and heat-inactivated *E. coli* 166. Moreover, both the ultrasonicated product and *E. coli* 166-derived exosomes exhibited inhibitory effects, though not as strong as those of live or heat-inactivated *E. coli* 166 (Fig. 4B).

To better understand the antiviral efficacy of *E. coli* 166 against ASFV, dose-response experiments using varying MOIs (25 and 100) and different preparations of bacterial lysates were conducted. The results showed that the antiviral activity of live *E. coli* 166 is dose dependent, with a significant difference observed between MOI = 25 and MOI = 100 (*P* < 0.01). Contrary to the live bacteria, the bacterial lysates, generated through sonication and heat-inactivation of intact bacterial cultures, did not exhibit a dose-dependent antiviral activity (Fig. 4C).

### The *E. coli* 166 strain exhibits characteristics similar to those of the nonpathogenic *E. coli* K-12 substrain MG1655

The genome of isolate *E. coli* 166 was sequenced, revealing a total length of 4,376,770 bp, closely resembling the reference genomes of other *E. coli* strains. *E. coli* 166 contains 5,630 genes, including six rRNA genes and 85 tRNA genes. Additionally, 1 prophage, 2 CRISPR sequences, and 11 genomic islands were identified. Figure S1A illustrated a circular representation of the *E. coli* 166 genome.

To assess the genomic similarity, we compared *E. coli* 166 with three commonly studied *E. coli* strains: the nonpathogenic K12, the probiotic strain EcN, and the pathogenic strains K88 and O157. LASTZ alignment indicated that *E. coli* 166 shares the highest similarity with nonpathogenic K12 (identical sites: 99.0%, pairwise identity: 98.8%, and reference coverage: 99.5%). The similarity between *E. coli* 166 and EcN shows identical sites: 97.0%, pairwise identity: 96.9%, and reference coverage: 88.9%. For pathogenic strains, the similarity between *E. coli* 166 and K88 is identical sites: 99.0%, pairwise identity: 98.8%, and reference coverage: 97.5%, while between *E. coli* 166 and O157, it is identical sites: 97.8%, pairwise identity: 97.6%, and reference coverage: 92.9% (Fig. S1B).

A comparison of virulence genes across O157, K88, EcN, K12, and *E. coli* 166 showed that *E. coli* 166 harbored a similar type but fewer virulence genes compared to the probiotic strain EcN (Table S2).

### Comparative analysis of cytokines repression in only ASFV-infected PAMs and 166-treated plus ASFV-infected PAMs

Since ASFV-eGFP replication was significantly reduced in 166-treated PAMs compared to untreated ones, we sought to investigate the immune response in different conditions. We tested and compared the expression levels cytokines in three groups: PAMs pretreated with *E. coli* 166 and infected with ASFV (166 treated), PAMs infected with ASFV only (positive control), and PAMs neither treated nor infected (negative control). Cytokine expressions were evaluated at 2, 5, 18, and 36 hpi, and the expression levels were calculated as fold changes.

**Fig 5:**
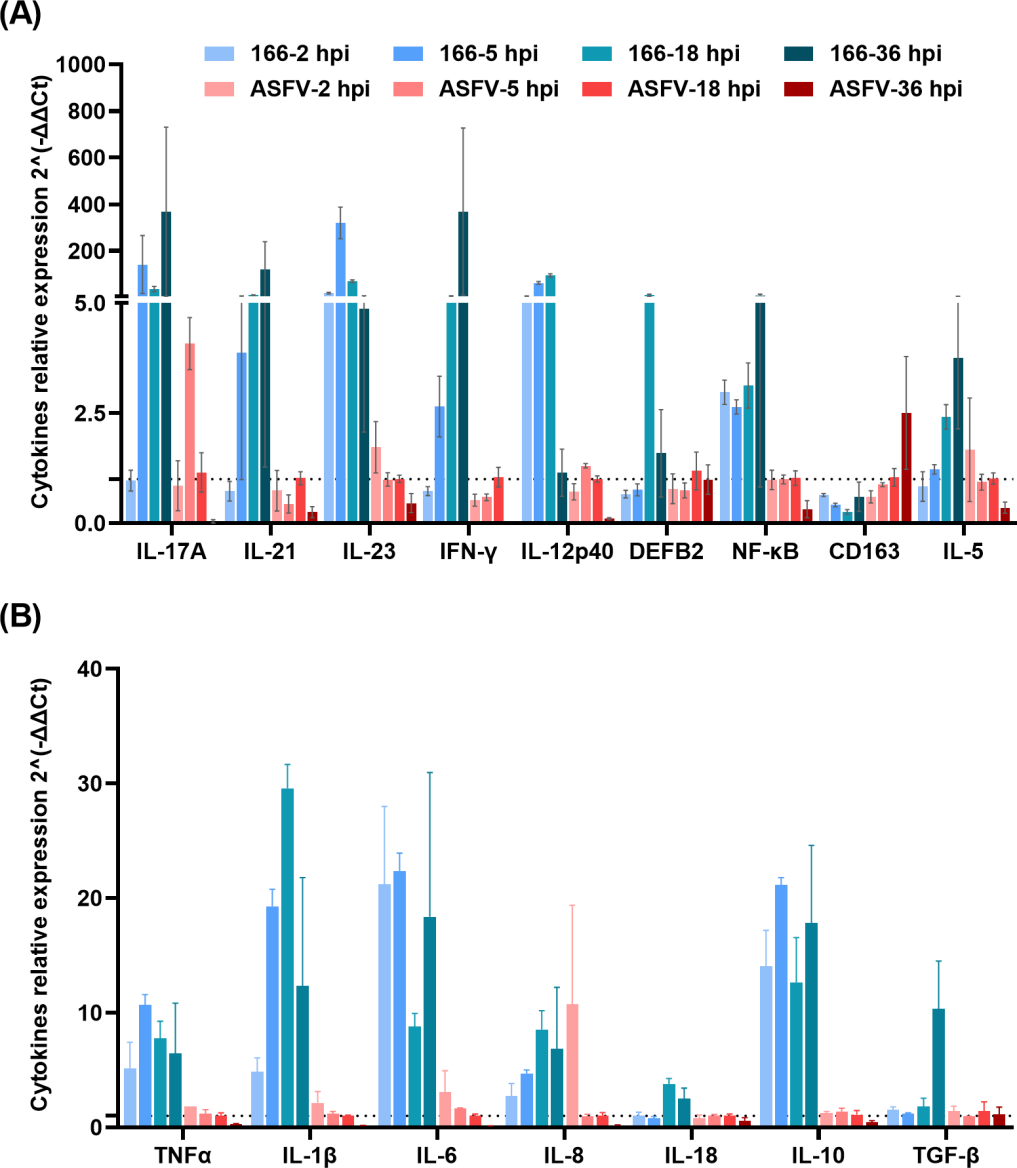
Comparative analysis of cytokines repression in only ASFV-infected PAMs and *E. coli* 166 treated and ASFV-infected PAMs. “ASFV-” represents PAMs only infected with ASFV, and “166-” represents *E. coli* 166-treated plus ASFV-infected PAMs. Cytokines expression is calculated by fold change, and *β*-actin is used as house keep gene. Cytokines expression is calculated at 2, 5, 18, and 36 hpi. The result is shown in fold change. For each timepoint, the data are calculated by Ct_cytokine_ − Ct*_β-_*_actin_ as the first ΔCt, then ΔΔCt = ΔCt_ASFV_ − ΔCt_negative_ or ΔΔCt = ΔCt_166_ − ΔCt_negative_ are calculated, respectively. If 2^−ΔΔCt^ is more than 1, means the gene is upregulated, otherwise if 2^−ΔΔCt^ is less than 1, means the gene is downregulated.

In the 166-treated PAMs, upregulation of NF-κB expression was observed at all timepoints (2, 5, 18, and 36 hpi), while NF-κB was downregulated at 2, 5, 18, and 36 hpi in PAMs infected only with ASFV. CD163 was consistently downregulated in 166-treated PAMs at all timepoints but was upregulated at 18 and 36 hpi in PAMs infected only with ASFV (Fig. 5A).

At 18 hpi, all cytokines were upregulated in 166-treated PAMs, except for CD163. This upregulation trend continued in 166-treated PAMs. By 36 hpi, most cytokines were downregulated in PAMs infected only with ASFV, except for CD163 and TGF-β, which remained upregulated. IL-10 and TGF-β, two anti-inflammatory cytokines, were upregulated in 166-treated PAMs at all four timepoints. Additionally, IL-17A, IL-21, IL-23, and IFN-γ were upregulated at 5, 18, and 36 hpi in 166-treated PAMs, while the expression trends of these four cytokines varied over time in PAMs infected only with ASFV (Fig. 5). The immune responses were different between only ASFV-infected PAMs and 166-treated plus ASFV-infected PAMs, upregulations of cytokines expression indicated the immunostimulatory effect of *E. coli* 166.

### Tn mutagenesis to screen for mutants with altered inhibition effect

To identify the possible bacterial products responsible for the inhibitory effect on ASFV replication, a genome-saturating mini-Tn5 transposon (Tn) mutant library containing Kna-resistant transformants was generated. A high-throughput screen of 5,000 *E. coli* 166 transposon mutants was conducted to identify genes involved in the inhibition of ASFV replication. While most mutants exhibited no significant change in inhibitory effect, a few mutants showed altered GFP/4',6-diamidino-2-phenylindole (DAPI) ratios, indicating potential loss or enhancement of the inhibition phenotype. Tn isolates with the same trends among four repeats on PAMs were selected for Tn sequence to identify the mutated gene. Through arbitrary PCR, mutated genes were identified (Table 1).

**TABLE 1:** List of genes identified from strains with altered inhibition effect

Gene	Product
aldA 1	Aldehyde dehydrogenase
aldA 2	Aldehyde dehydrogenase
araD 1	Ribulokinase
araD 2	Ribulokinase
argE 1	Acetylornithine deacetylase
argE 2	Acetylornithine deacetylase
argE 3	Acetylornithine deacetylase
clsA	Cardiolipin synthase
dksA^*a*^	RNA polymerase-binding transcription factor
efeO^*a*^	Iron transporter EfeO
epcB	Ectoine hydroxylase
esiB 1^*a*^	Stress response protein EsiB
esiB 2^*a*^	Stress response protein EsiB
fadK^*a*^	Acyl-CoA synthetase
fdoG 1	Formate dehydrogenase-O subunit alpha
fdoG 2	Formate dehydrogenase-O subunit alpha
fdoG 3	Formate dehydrogenase-O subunit alpha
fdoG 4	Formate dehydrogenase-O subunit alpha
glpR 1	Glycerol-3-phosphate regulon repressor
glpR 2	Glycerol-3-phosphate regulon repressor
glpR 3^*a*^	Glycerol-3-phosphate regulon repressor
HT1_chunhua_01329^*a*^	Outer membrane porin PhoE
HT1_chunhua_01330^*a*^	Outer membrane porin PhoE
HT1_chunhua_03622	4-Hydroxy-tetrahydrodipicolinate reductase
HT1_chunhua_04192	Glutathione S-transferase
HT1_chunhua_04199	Ribonuclease BN
HT1_chunhua_05250	Beta-galactosidase
malQ 2	4-Alpha-glucanotransferase
mrcB 2	Penicillin-binding protein 1B
nagB 4	Glucosamine-6-phosphate deaminase
nagB 5	Glucosamine-6-phosphate deaminase
nrfB	Nitrite reductase
oppA 1	Oligopeptide transport ATP-binding protein
ppk	Polyphosphate kinase
purH 1	Bifunctional purine biosynthesis protein
rhmR	L-rhamnose operon transcriptional repressor
rsd^*a*^	Regulator of sigma D
thiC	Thiamine biosynthesis protein ThiC
thiE 1	Thiamine-phosphate pyrophosphorylase
thiE 2	Thiamine-phosphate pyrophosphorylase
tldD 1	Murein peptide amidase
tldD 2	Murein peptide amidase
ygbF^*a*^	2-C-methyl-D-erythritol 4-phosphate cytidylyltransferase
yibH	Uncharacterized membrane protein YibH
yihV 1	Sugar efflux transporter
yihV 2	Sugar efflux transporter

^*a*^ Represents that genes or locus tag could not be deleted.

**Fig 6:**
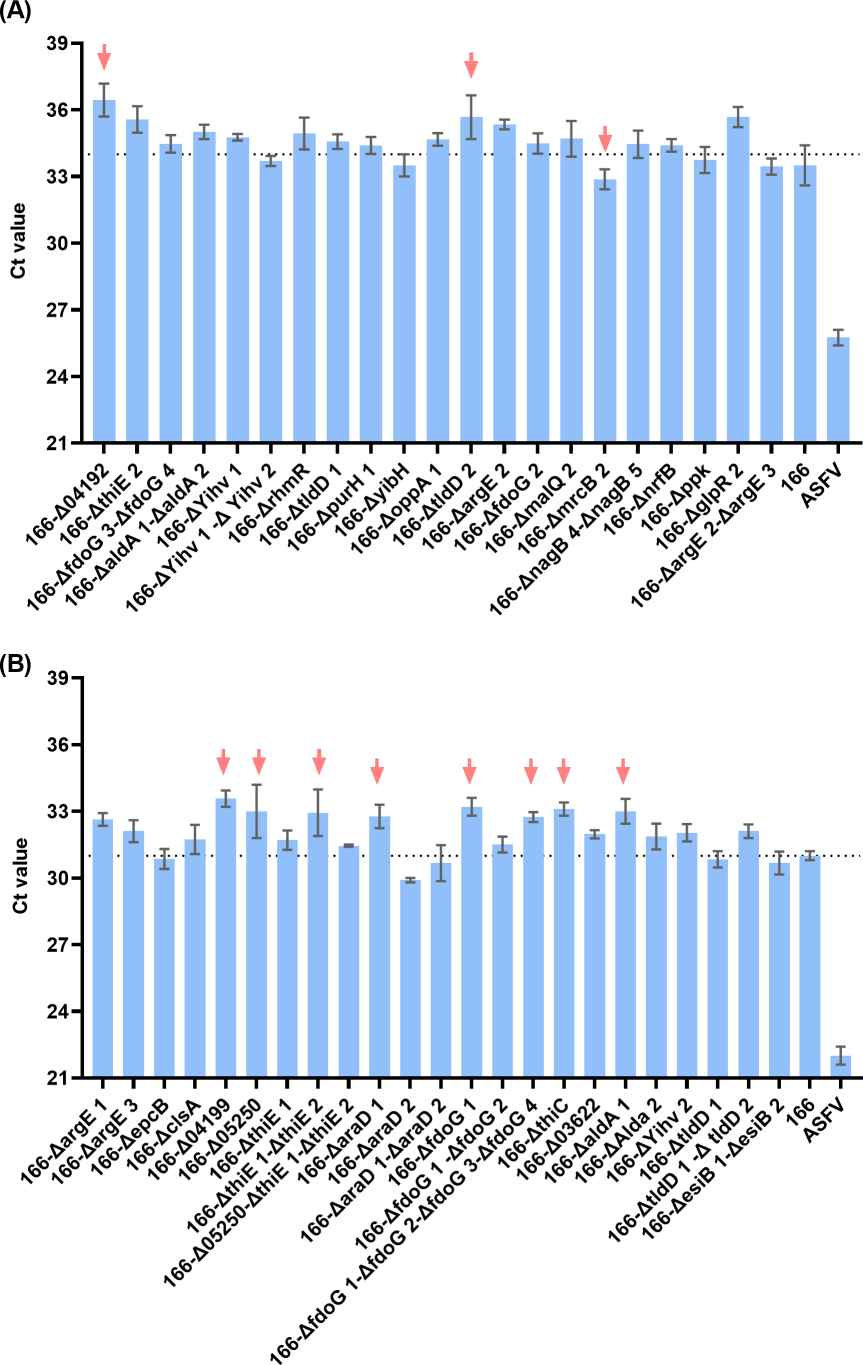
Evaluation of inhibition effect on ASFV of genetic modified *E. coli* 166 strains. (**A and B**) Inhibition effect of genetic modified *E. coli* 166 was evaluated, ΔCt value = Ct_genetic modified_ − Ct_166_, if it exceeded 1.5, it was highlighted with red arrow.

Through the lambda red recombineering technique, 44 genetic modified *E. coli* 166 strains were successfully constructed, while nine genes or locus tag (*dskA*, *efeO*, *esiB 1*, *esi B 2*, *fadK*, *glpR 3*, *01329–01330*, *rsd*, and *ygbF*) could not be deleted (Table 1). The inhibition effect on the replication of ASFV of the respective strain was evaluated. In general, the inhibition effect was consistent with the observation of the high-throughput screen results, though the differences were not statistically significant. According to the ΔCt value (Ct_genetic modified_ − Ct_166_), most of the modified strains have Ct values similar to those of *E. coli* 166. A ΔCt exceeding 1.5 or −1.5 was highlighted (Fig. 6). Deletion of a specific gene failed to alter the inhibition phenotype of *E. coli* 166, indicating that genes selected may not be responsible for the inhibition, or it is not a single gene or a single product that plays the inhibition role.

## DISCUSSION

The gut microbiota consists of millions of bacteria, although not all are culturable. This complex community plays a crucial role in various physiological processes, including digestion, immune regulation, and maintaining overall health. The diversity and balance of gut microbiota are essential for maintaining homeostasis and preventing diseases. The present study isolates nearly 100 different isolates from wild boar intestinal content through bacterial culturomics. Most importantly, we found that one isolate designated as *E. coli* 166 is capable of stimulating the NF-κB pathway and inhibiting the replication of different ASFV isolates.

Our results indicate that both live and heat-inactivated *E. coli* 166 significantly inhibit ASFV replication. Notably, the inhibitory effect of live *E. coli* 166 is more pronounced at a higher MOI than at a lower MOI. Although exosomes and ultrasonicated products of *E. coli* 166 also show antiviral activity, their effects are notably weaker than those of the intact bacteria. Furthermore, no significant difference in the inhibitory effect was observed between different doses of ultrasonicated and heat-inactivated bacterial lysates. These findings suggest that *E. coli* 166 may inhibit ASFV without requiring direct interaction with PAMs, and the active antiviral components likely comprise a complex mixture of secreted metabolites and lysis products. The diminished efficacy of exosomes and ultrasonicated products implies that these isolated components do not represent the full spectrum of antiviral factors presented in live or heat-inactivated bacteria.

Exosomes derived from *Enterococcus faecium* C171 have been shown to promote pro-inflammatory responses, enhance immunity, and maintain immune homeostasis (15). In contrast, exosomes from *Fusobacterium nucleatum* activate the TLR2-MyD88-NF-κB signaling pathway, thereby inducing pro-inflammatory effects and modulating innate immune responses (21). The relatively weak antiviral effects of the ultrasonic lysates may result from the disruptive nature of ultrasonic treatment, which can compromise critical active components and molecular structures. Previous studies indicate that ultrasonic processing can degrade bioactive molecules (22–24), thereby reducing their capacity to inhibit viral replication. Thus, while ultrasonic lysates may possess certain antibacterial properties, the conditions of ultrasonic treatment may limit their overall efficacy against ASFV.

CD163 is considered one of the most likely receptors for ASFV and could serve as a potential target to inhibit viral uptake (25). The upregulation of CD163 correlates with ASFV’s interaction with this receptor during viral entry and may contribute synergistically to the process of ASFV infection in cell lines (26, 27). However, genetically modified pigs deficient in CD163 do not exhibit resistance to infection with the Georgia 2007/1 strain, suggesting that the host’s immune system and viral pathogenesis are more complex than previously assumed (28). Regardless of this, downregulation of CD163 expression is observed in the early time (2 and 5 hpi) for both 166-treated PAMs and not treated PAMs; however, CD163 is upregulated for PAMs infected only with ASFV at both 18 and 36 hpi, while still keep downregulated in 166-treated PAMs. This might be one of the reasons lower viral replications were observed in the 166 treated PAMs. Therapeutics that block CD163 or modulate its expression could reduce ASFV infection levels by preventing the virus from effectively entering host cells.

ASFV inhibits the activation of NF-κB, a key transcription factor in the host immune response, through several strategies. The ASFV gene A238L binds to the p65 (RelA) subunit of NF-κB, while proteins such as pD345L and I10L target the IκB kinase (IKK) complex to suppress the host’s inflammatory response (29). The MGF360 and MGF530 multigene families also interfere with the NF-κB pathway, aiding in evading the host immune system (30). Thus, the NF-κB signaling pathway is a crucial immunomodulatory target in controlling ASFV (31). *E. coli* 166 activates IRF and NF-κB signaling pathways with J774-Dual cells, and when the NF-κB was tested by qPCR in the PAMs, upregulation of NF-κB expression was observed at all the timepoints (2, 5, 18, and 36 hpi) for *E. coli* 166-treated PAMs, while it was downregulated at 2, 5, 18, and 36 hpi for PAMs only infected with ASFV. The treatment with *E. coli* 166 can activate the NF-κB pathway, thereby counteracting the inhibitory effect of ASFV on NF-κB activation.

High levels of IFN-γ, a key cytokine in promoting Th1 responses and activating macrophages, have been associated with better control of ASFV (32), and the upregulation of IFN-γ is observed at 5, 18, and 36 hpi in the *E. coli* 166-treated PAMs. Additionally, there was a significant upregulation of anti-inflammatory cytokines IL-10 and TGF-β across all time points in the *E. coli* 166-treated PAMs. Although upregulation is observed in PAMs infected only with ASFV, the fold change in *E. coli* 166 treated PAMs is higher than only ASFV-infected PAMs, indicating an enhanced anti-inflammatory response that supports tissue repair and immune regulation (33). The upregulation of Th17-associated cytokines IL-17A, IL-21, and IL-23 further supports the role of *E. coli* 166 in enhancing mucosal immunity and promoting a robust immune defense against ASFV (32).

In our study, we performed Tn mutagenesis on *E. coli* 166 to identify the genetic factors responsible for its inhibitory effect on ASFV replication. Although a library of Tn mutants was constructed, none of the gene deletions led to a significant loss of the inhibition phenotype. This lack of a clear phenotype change suggests the presence of genetic redundancy within the *E. coli* 166 genome, where multiple genes or pathways can compensate for the loss of a single gene, thereby maintaining the bacterium’s antiviral properties. Gene redundancy is a well-documented phenomenon in bacterial genomes, where several genes perform overlapping functions (34). This genetic robustness ensures that critical functions are preserved even in the event of gene loss or mutation. In the case of *E. coli* 166, the redundancy likely involves multiple pathways or genes that contribute to the suppression of ASFV replication. This redundancy complicates the identification of specific genetic determinants responsible for the observed phenotype but also highlights the evolutionary advantage of such a robust system in maintaining essential functions under various environmental pressures (35, 36). Further research is needed to explore redundant pathways and assess whether simultaneous knockout of multiple genes affects the antiviral activity of *E. coli* 166. Investigating the released compounds and the collective roles of candidate genes, possibly through combined deletions, may provide deeper insights into the genetic basis of ASFV inhibition.

These findings underscore the potential of *E. coli* 166 as an effective intervention to enhance immune responses and mitigate ASFV infection. It is intriguing to consider whether ASFV variants resistant to the inhibitory effects of *E. coli* 166 could emerge. Although no such variants were observed in the current study, future experiments may involve serial passaging of ASFV in co-culture with *E. coli* 166 and PAMs under conditions conducive to viral adaptation or the screening of ASFV isolates from clinical settings. Comparative genomic analyses of resistant and non-resistant ASFV isolates, along with investigations into host response variations, could further elucidate the mechanisms of resistance. Additionally, future studies in animal models are essential to evaluate the *in vivo* efficacy of *E. coli* 166 as a therapeutic agent of ASFV, particularly in the context of oral administration and microbiota modulation. Understanding the mechanisms of action and the *in vivo* efficacy of probiotic *E. coli* 166 in swine models is crucial for optimizing probiotic strains with enhanced antiviral properties. This knowledge could significantly contribute to more effective biocontrol strategies against ASFV.

## MATERIAL AND METHODS

### Cell culture and virus

PAMs were isolated from the bronchoalveolar lavage fluid of healthy 4-week-old, specific pathogen-free piglets and cultured in RPMI-1640 medium supplemented with 10% fetal bovine serum, 100 U/mL penicillin, and 50 mg/mL streptomycin. The ASFV-eGFP strain (HLJ/18–6GD) was developed and preserved at the Harbin Veterinary Research Institute (HVRI) (7). All experiments with ASFV-eGFP, genotype II virulent virus HLJ/18, genotype II moderately virulent variant HLJ/HRB1/20, and genotype I low virulent virus SD/DY-I/21 were conducted within the biosafety level 3 laboratory in the HVRI of the Chinese Academy of Agricultural Sciences approved by the Ministry of Agriculture and Rural Affairs.

### Fecal sampling and processing

Samples of gastrointestinal contents from the duodenum, ileum, cecum, and colon were collected immediately post-slaughter from a healthy wild boar originating in the Greater Khingan Mountains, Jiagedaqi City. This wild boar was screened for common porcine viruses, such as ASFV, porcine reproductive and respiratory syndrome virus, classical swine fever virus, pseudorabies virus, and porcine circovirus type 2, using PCR testing.

The fecal samples were processed following a previously established method (8). Each sample was diluted in 1× phosphate-buffered saline (PBS) from concentrations of 10⁻¹ to 10⁻⁹, and 200 µL from each dilution was spread onto brain heart infusion, de Man, Rogosa, and Sharpe, and yeast extract, casitone, and fatty acids agar plates. Duplicate plates were then incubated under both aerobic and anaerobic conditions at 37℃ for up to 72 h.

### Isolation and identification of bacteria

Colonies were subcultured on fresh agar plates until pure isolates were obtained. Each isolate was then suspended in 500 µL of sterile PBS and stored at −20℃ until DNA extraction. For bacterial genomic DNA extraction, 250 µL of each PBS suspension was processed using the Chelex-based Instagene Matrix (Bio-Rad), according to the manufacturer’s protocol. The complete 16S rDNA gene was amplified with universal primers 27F (5′-AGAGTTTGATCCTGGCTCAG-3′) and 1492R (5′-CGGTTACCTTGTTACGACTT-3′) (37), and Sanger sequencing was performed for identification. Sequences were compared using BLAST in the Ribosomal Database (https://blast.ncbi.nlm.nih.gov/Blast.cgi?PROGRAM=blastn&PAGE_TYPE=BlastSearch&LINK_LOC=blasthome). Isolates identified as the same genus were further analyzed through ERIC-PCR (38) using primers ERIC-1F (5′-ATGTAAGCTCCTGGGGATTCAC-3′) and ERIC-2R (5′-AAGTAAGTGACTGGGGTGAGCG-3′) (39). From each unique fingerprint pattern, one representative isolate was chosen for further experiments.

### High-throughput screening of the immunoregulatory effect of wild boar bacterial isolates on J774-Dual reporter cells

The J774-Dual cells, developed from the macrophage-like mouse cell line J774.1, were engineered to stably express two inducible reporter constructs. The activation of the NF-κB and IRF signaling pathways was monitored through the detection of secreted embryonic alkaline phosphatase (SEAP) and luciferase activities in the culture supernatant, respectively.

Experiments using J774-Dual cells were conducted according to the manufacturer’s guidelines. Cells were prepared at a concentration of 2.8 × 10⁵ cells/mL and plated in 96-well plates. Live bacteria were subsequently added to the wells containing cells. To serve as positive controls, Pam3CSK4 (10 ng/mL)was used for NF-κB pathway activation, and 2’3’-cGAMP (1 µg/mL) for IRF pathway activation. After 24 h of stimulation, the supernatant was collected for analysis.

To assess NF-κB activation, 30 µL of the cell supernatant was placed in a 96-well plate and mixed with 170 µL of Quanti-Blue reagent (InvivoGen, France). The plate was incubated at 37°C with 5% CO_2_ for 4 h, after which absorbance at 650 nm was read on a microplate reader to measure SEAP levels as an indicator of NF-κB activation. For IRF pathway analysis, luciferase activity was quantified using an LB 960 chemiluminescence detector.

### Evaluating the cytotoxicity of isolates on PAMs

Cytotoxicity of the isolates was evaluated using the CCK-8 (Dojindo, Japan), following the manufacturer’s protocol. Briefly, PAMs were plated in a 96-well plate and incubated at 37°C with 5% CO_2_ for 12 h. Subsequently, 100 µL of bacterial suspension in cell culture medium, at an MOI of 50, was added to the wells. The plate was incubated at 37°C with 5% CO_2_ for both 4 and 72 h. Then, 10 µL of CCK-8 solution was added to each well, followed by an additional 2 h incubation at 37°C. Absorbance was read at 450 nm using an ELx808 microplate reader to assess cell viability.

### Evaluating the inhibitory effect of bacteria on ASFV replication in PAMs

PAMs were seeded into a 48-well plate (3 × 10^7^ cells/plate), and after the cells were completely adherent, fresh cultured bacteria (MOI = 50) were added and incubated at 37°C with 5% CO_2_ for 2 h, and then ASFV isolates (HLJ/18–6GD, MOI = 0.1) was inoculated for 2 h. The cells were washed with PBS for three times, then RPMI 1640 medium was added and incubated at 37°C with 5% CO_2_. This timepoint is 0 hpi. The supernatant was harvested at 48 hpi.

ASFV genomic DNA was extracted from cell culture supernatant. qPCR recommended by WOAH was carried out by using a QuantStudio 5 system (Applied Biosystems, USA) as described previously (40). ASFV replication level is represented by the Ct value; a higher Ct value indicates a lower quantity of ASFV genomes. A ΔCt of 3.3 corresponds to 10 times the genomic difference.

### Characterization of *E. coli* 166

The whole genome of *E. coli* 166 was sequenced using Wtdbg2 third-generation sequencing technology, provided by Shanghai OE Biotechnology Co., Ltd., China. The *E. coli* 166 genome was compared to four complete *E. coli* strains: a nonpathogenic *E. coli* str.K-12 substr. MG1655 (K12; NC_000913) (41), a probiotic strain *E. coli* (42) (EcN; GCA_000714595.1) (42), a pathogenic *E. coli* UMNK88 (K88; NC_017641) (43), and a pathogenic *E. coli* O157:H7 str. EC4115 (O157; NC_011353) (44). Initially, the genomes of *E. coli* strains K12, EcN, K88, O157, and *E. coli* 166 were compared using Geneious Prime software. Subsequently, the genomes of K12, EcN, K88, and O157 were aligned with that of *E. coli* 166 using LASTZ alignment. Virulence genes from *Escherichia* were obtained from the Virulence Factor Database (VFDB) (http://www.mgc.ac.cn/VFs/) and compared across K12, EcN, K88, and O157 against those in *E. coli* 166.

### Evaluating the inhibitory effect of *E. coli* 166 on different ASFVs replication in PAMs

PAMs were seeded into a 48-well plate (3 × 10^7^ cells/plate), and after the cells were completely adherent, fresh cultured bacteria (MOI = 50) were added to incubate for 2 h. Subsequently, four ASFV isolates were inoculated at an MOI of 0.1, including the genotype II virulent strain HLJ/18, the genotype II naturally mutated moderately virulent strain HLJ/HRB1/20, the genetically modified low-virulent strain HLJ/18–6GD, and the genotype I low-virulent strain SD/DY-I/21, respectively, followed by a 2 h incubation. Then, the cells were washed three times with PBS, and the RPMI 1640 medium was added and incubated at 37°C with 5% CO_2_, and the supernatant was harvested at 48 hpi. Viral DNA extraction and quantification were performed as described in the section Evaluating the inhibitory effect of bacteria on ASFV replication in PAMs.

### Evaluating the inhibitory effect of different forms of *E. coli* 166 on ASFV replication in PAMs

The experimental setups were divided into two groups: (i) direct interaction, where *E. coli* 166 was added directly to PAMs, and (ii) indirect interaction, where *E. coli* 166 was placed in the transwell insert to prevent direct contact with PAMs. The transwell coculture system was used to assess whether direct interaction between PAMs and *E. coli* 166 is necessary for its inhibitory effect. The PAMs were grown in the 24-well plate (3 × 10^7^ cells/plate) equipped with permeable supports (polycarbonate membrane inserts with a pore size of 0.4 µm, Corning); this setup allowed us to assess if the inhibition was dependent on direct contact or if it was mediated solely by the metabolic products or lysis components released by *E. coli* 166. Wells containing a transwell insert, referred to as the “transwell” group, represented conditions where *E. coli* 166 was added in the transwell insert, and thus did not have direct contact with PAMs. Wells without a transwell insert, referred to as the “direct” group, represented conditions where *E. coli* 166 was added into the well directly, allowing direct interaction between *E. coli* 166 and PAMs. HLJ/18–6GD was inoculated in both the “transwell” and “direct” wells. As a control, cells infected only with HLJ/18–6GD without *E. coli* 166 treatment were used as the “positive” group.

We wondered whether dead *E. coli* 166 or 166 culture medium had the inhibitory effect or not. To demonstrate that different forms of *E. coli* 166 (heat-inactivated *E. coli* 166, ultrasonication *E. coli* 166, and exosomes of *E. coli* 166) were produced. Basically, fresh *E. coli* 166 culture was centrifuged, the pellet was washed three times with PBS, then the pellet was resuspended and further treated by boiling (85°C for 30 minutes) or ultrasonicated till the resuspension became transparent. Exosomes were extracted from *E. coli* 166 as described previously (45, 46). Heat-inactivated *E. coli* 166, ultrasonicated *E. coli* 166, and exosomes of *E. coli* 166 were then filtered using 0.45 µm filter. The inhibitory effect of different forms of *E. coli* 166 was evaluated. The rest of the procedure was performed as described in the section Evaluating the inhibitory effect of bacteria on ASFV replication in PAMs.

We then evaluated the dose-response relationship, different MOIs of live *E. coli* 166, and dilutions of bacterial lysates obtained through sonication and heat inactivation of intact *E. coli* 166 cultures. The rest of the procedure was performed as described in the section Evaluating the inhibitory effect of bacteria on ASFV replication in PAMs.

### Measurement of cytokine expression

PAMs were seeded into a 12-well plate (3 × 10^7^ cells/plate). The rest is as same as described in The *E. coli* 166 strain exhibits characteristics similar to those of the nonpathogenic *E. coli* K-12 substrain MG1655, except that the supernatant was harvested at 0, 5, 18, and 36 hpi at 37°C with 5% CO_2_. Subsequently, 600 µL of TRIzol was added to each well, and the plates were immediately frozen at −80°C until RNA extraction. RNA was extracted by using the TRIzol method, following the manufacturer’s instructions.

RNA concentration and purity were evaluated using BioDrop. cDNA was synthesized using the FastQuant reverse transcription kit with gDNase (Vazyme Biotech Co., China) in accordance with the manufacturer’s protocol. β-actin served as the internal reference gene. Quantitative real-time PCR was conducted to determine the expression levels of 16 porcine genes associated with Th1, Th2, and Th17 immune responses, as previously reported (47). Primers were used at a final concentration of 0.5 µM per reaction, with sequences provided in Table 2. Relative gene expression was analyzed using the 2^−ΔΔCt^ method as described in reference (48).

**TABLE 2:** List of genes and sequences of the primers used for cytokines checked in qPCR analysis

Gene name	Forward primer (5′ → 3′)	Reverse primer (5′ → 3′)	Accession number
*TNFα*	CCTCTTCTCCTTCCTCCTG	CCTCGGCTTTGACATTGG	X57321
*IL-1β*	GGCCGCCAAGATATAACTGA	GGACCTCTGGGTATGGCTTTC	NM_214055
*IL-5*	TGGTGGCAGAGACCTTGACA	CCATCGCCTATCAGCAGAGTT	NM_214205.1
*IL-6*	TGGCTACTGCCTTCCCTACC	CAGAGATTTTGCCGAGGATG	NM_214399
*IL-8*	TTCGATGCCAGTGCATAAATA	CTGTACAACCTTCTGCACCCA	AB057440
*IL-10*	CAGATGGGCGACTTGTTG	ACAGGGCAGAAATTGATGAC	L20001
*IL-12p40*	GGAGTATAAGAAGTACAGAGTGG	GATGTCCCTGATGAAGAAGC	U08317
*IL-17A*	CTCTCGTGAAGGCGGGAAT	TCCTCAGTTTTTGGGCATCCT	NM_001005729
*IL-18*	AGGGACATCAAGCCGTGTTT	CGGTCTGAGGTGCATTATCTGA	EU118362.1
*IL-21*	CTGCCTGATGGTCATCTTCTCA	AGGCGATCTTGTCCTTGGAA	NM_214415
*IL-23*	GCTTGCAAAGGATCCACCAA	GGCTCCCCTGTGAAAATGTC	NM_001130236.1
*IFN-γ*	CAAAGCCATCAGTGAACTCATCA	TCTCTGGCCTTGGAACATAGTCT	X53085
*TGF-β*	TCAGGCTTACCATTGCTTGTTCAG	CTCGCCAAACCTCTCCAAATCG	NM_001038639
*DEFB2*	CTGTCTGCCTCCTCTCTTCC	CAGGTCCCTTCAATCCTGTT	NM_214442
*NF-κB*	CTCGCACAAGGAGACATGAA	ACTCAGCCGGAAGGCATTAT	DQ834921
*CD163*	GCCTGTCTCATCGCATTCCT	GGATTTAGCATATCCGTTTCATCTG	NM_213976.1
*β-actin*	CAGGTCATCACCATCGGCAACG	GACAGCACCGTGTTGGCGTAGAGGT	U07786

### Tn mutagenesis to screen the mutants with altered inhibition effect

The bacterial strains were routinely cultured on solid Luria-Bertani (LB) agar or in liquid LB medium supplemented with appropriate antibiotics as required at 37°C. The antibiotics used in the present study were ampicillin (100 µg/mL), kanamycin (Kan; 50 µg/mL), nalidixic acid (Nal; 30 µg/mL), or chloramphenicol (25 µg/mL).

To screen the genetic factors involved in inhibiting ASFV replication, a Tn (pUTmini-Tn5km2, KmR) library based on the *E. coli* 166 was constructed according to previously reported (49). Aliquots containing ~300–400 mutants were cultured on LB agar plates supplemented with Nal and Kan to select for mutants (49).

Fresh bacteria were prepared by inoculating a single colony into LB medium (supplemented with Nal and Kan) and culturing overnight at 37°C. The following day, the culture was reseeded in fresh LB medium, and once the parental *E. coli* 166 reached an OD_600_ of 1.0, an equal volume of each bacterium was added to the PAMs. PAMs were seeded into a 96-well plate (3 × 10^7^ cells/plate). Once the cells were completely adherent, fresh cultured bacteria were added and incubated for 2 h. HLJ/18–6GD was then inoculated at an MOI of 0.1 and incubated for an additional 2 h. The cells were washed three times with PBS, and RPMI 1640 medium was added. Plates were incubated at 37°C with 5% CO_2_ for 36 h.

Each experiment included three groups: 166-treated and ASFV-infected cells as the experimental group, ASFV-infected cells without 166 treatment as the positive control, and uninfected cells as the negative control. After incubation, the supernatant was removed, and cells were fixed with 4% formalin for 20 minutes at room temperature. Cells were then washed with PBS and stained with Hoechst for 15 minutes. Then, the Hoechst stain was discarded, and 100 µL of PBS was added to each well. Fluorescence images were captured using a fluorescence microscope (GFP for ASFV-infected cells and DAPI for total cells), with 80% coverage per well. The GFP/DAPI ratio was used to assess ASFV replication, and isolates with higher relative GFP/DAPI ratios were selected for further analysis. For isolates showing inhibition effect, the inhibition effect was confirmed on PAMs with 48-well plates, as described in the section Evaluating the inhibitory effect of bacteria on ASFV replication in PAMs.

Arbitrary PCR was used to map Tn insertion sites (49) for bacteria that showed inhibition potential, and the lambda-red recombineering technique was used for gene-deleting purposes (50). Then, the inhibitory effect of genetically modified *E. coli* 166 strains was evaluated in PAMs with 48 well plates, as described in section Evaluating the inhibitory effect of bacteria on ASFV replication in PAMs.

### Statistical analysis

Statistical analysis was conducted using GraphPad Prism. One-way Analysis of Variance (ANOVA) was employed to assess the immunoregulatory effects of wild boar bacterial isolates, the inhibitory effects of *E. coli* isolates 71 and 166 on ASFV replication, and the inhibition of ASFV replication by different forms of *E. coli* 166. A paired *t*-test was used to analyze the inhibitory effect of *E. coli* 166 on different ASFV strains. Statistical significance was defined as *P < 0.05, **P < 0.01, ***P < 0.001, and ****P < 0.0001, and ns refers to no significance.

## Data Availability

Data will be made available on request.

## SUPPLEMENTAL MATERIAL

Supplemental material spectrum.03009-24-s0001.pdf Fig. S1; Tables S1 and S2.

ASM does not own the copyrights to Supplemental Material that may be linked to, or accessed through, an article. The authors have granted ASM a non-exclusive, world-wide license to publish the Supplemental Material files. Please contact the corresponding author directly for reuse.
