# Supplementary material for: Identification of Escherichia coli 166 isolate as an effective inhibitor of African swine fever virus replication
Source: Microbiol Spectr. 2025 Feb 26;13(4):e03009-24. doi: 10.1128/spectrum.03009-24 (PMC11960076; doi:10.1128/spectrum.03009-24)
Supplement: Supplemental material — Fig. S1; Tables S1 and S2. [file spectrum.03009-24-s0001.pdf]

Table S1. 16S rDNA sequences of four isolates 114, 318, 71 and 166.

Table S2. Comparison of the virulence genes among O157, K88, EcN, K12 and the *E. coli* 166 genomes.

Figure S1. Genome circle of *E. coli* 166 and LASTZ dot plots comparison. (A) The concentric circles show from outside to inside: 1st circle, Genomic sequence information. 2rd circle, Coding sequences (CDS) and non-coding RNA regions (rRNA, tRNA) from the reference genome. The outer layer represents the positive strand, and the inner layer represents the negative strand. 3rd circle, GC skew curve with a 2,000 bp sliding window. The dashed line represents GC skew = 0. 4th circle, GC content curve with a 2,000 bp sliding window. The dashed line represents the average GC content of the reference genome. **(B)** LASTZ (Large-Scale Genome Alignment Tool) analysis compare the consensus in genomes among *E. coli* 166 and other four *E. coli* reference strains.

Table S1. 16S rDNA sequences of four isolates 114, 318, 71 and 166.

>71

ACGGCCTGGGCGGCAGCTACACATGCAGTCGAACGGTAACAGGAAGCAGCTTGCTGCTTCGCTGACG
AGTGGCGGACGGGTGAGTAATGTCTGGGAAACTGCCTGATGGAGGGGGATAACTACTGGAAACGGTA
GCTAATACCGCATAACGTCGCAAGACCAAAGAGGGGGACCTTCGGGCCTCTTGCCATCGGATGTGCCC
AGATGGGATTAGCTAGTAGGTGGGGTAACGGCTCACCTAGGCGACGATCCCTAGCTGGTCTGAGAGGA
TGACCAGCCACACTGGAACTGAGACACGGTCCAGACTCCTACGGGAGGCAGCAGTGGGGAATATTGC
ACAATGGGCGCAAGCCTGATGCAGCCATGCCGCGTGTATGAAGAAGGCCTTCGGGTTGTAAAGTACTT
TCAGCGGGGAGGAAGGGAGTAAAGTTAATACCTTTGCTCATTGACGTTACCCGCAGAAGAAGCACCG
GCTAACTCCGTGCCAGCAGCCGCGGTAATACGGAGGGTGCAAGCGTTAATCGGAATTACTGGGCGTAA
AGCGCACGCAGGCGGTTTGTTAAGTCAGATGTGAAATCCCCGGGCTCAACCTGGGAACTGCATCTGAT
ACTGGCAAGCTTGAGTCTCGTAGAGGGGGGTAGAATTCCAGGTGTAGCGGTGAAATGCGTAGAGATCT
GGAGGAATACCGGTGGCGAAGGCGGCCCCCTGGACGAAGACTGACGCTCAGGTGCGAAAGCGTGGG
GAGCAAACAGGATTAGATACCCTGGTAGTCCACGCCGTAAACGATGTCGACTTGGAGGTTGTGCCCTT
GAGGCGTGGCTTCCGGAGCTAACGCGTTAAGTCGACCGCCTGGGGAGTACGGCCGCAAGGTTAAAAC
TCAAATGAATTGACGGGGGCCCGCACAAGCGGTGGAGCATGTGGTTTAATTCGATGCAACGCGAAGA
ACCTTACCTGGTCTTGACATCCACAGAACTTTCCAGAGATGGATTGGTGCCTTCGGGAACTGTGAGAC
AGGTGCTGCATGGCTGTCGTCAGCTCGTGTTGTGAAATGTTGGGTTAAGTCCCGCACGAGCGCACCCT
ATCCTTTGTTGCCAGCGGTCCGCGGGACTCAAGAGACTGCCAGTGATAACCTGGAAGAAGGTGGGGA
AGACCGTCAGTCATCATGGCCTTACAACACAGGGCCTACACACTGCTAATGGGCATACAAAGAGAAGC
GCACCTCCGAAAGAAGCGGACCTCATTATGCGTCCTCTATAATCCCGATGA

>318

GCGGGCTGGGCGGGAGGCTACACATGCAGTCGAACGGTAACAGGAAGCAGCTTGCTGCTTCGCTGAC
GAGTGGCGGACGGGTGAGTAATGTCTGGGAAACTGCCTGATGGAGGGGGATAACTACTGGAAACGGT
AGCTAATACCGCATAACGTCGCAAGACCAAAGAGGGGGACCTTCGGGCCTCTTGCCATCGGATGTGCC
CAGATGGGATTAGCTAGTAGGTGGGGTAACGGCTCACCTAGGCGACGATCCCTAGCTGGTCTGAGAGG
ATGACCAGCCACACTGGAACTGAGACACGGTCCAGACTCCTACGGGAGGCAGCAGTGGGGAATATTG
CACAATGGGCGCAAGCCTGATGCAGCCATGCCGCGTGTATGAAGAAGGCCTTCGGGTTGTAAAGTACT
TTCAGCGGGGAGGAAGGGAGTAAAGTTAATACCTTTGCTCATTGACGTTACCCGCAGAAGAAGCACC
GGCTAACTCCGTGCCAGCAGCCGCGGTAATACGGAGGGTGCAAGCGTTAATCGGAATTACTGGGCGTA
AAGCGCACGCAGGCGGTTTGTTAAGTCAGATGTGAAATCCCCGGGCTCAACCTGGGAACTGCATCTG
ATACTGGCAAGCTTGAGTCTCGTAGAGGGGGGTAGAATTCCAGGTGTAGCGGTGAAATGCGTAGAGAT
CTGGAGGAATACCGGTGGCGAAGGCGGCCCCCTGGACGAAGACTGACGCTCAGGTGCGAAAGCGTG
GGGAGCAAACAGGATTAGATACCCTGGTAGTCCACGCCGTAAACGATGTCGACTTGGAGGTTGTGCCC
TTGAGGCGTGGCTTCCGGAGCTAACGCGTTAAGTCGACCGCCTGGGGAGTACGGCCGCAAGGTTAAA
ACTCAAATGAATTGACGGGGGCCCGCACAAGCGGTGGAGCATGTGGTTTAATTCGATGCAACGCGAA
GAACCTTACCTGGTCTTGACATCCACAGAACTTTCCAGAGATGGATATGTGCCTTCGGGACTGTGAGA
CAGTGCTGCATGGCTGTCGTCAGCTCGTGTGTGAAATGTGGGTAAGTCCGCACGAGCGCACCCTTATC
TTTGTTGCAGCGGTCGGCGGGACTCAAGGGACTGCATGATAACTGGAGGAGGTGGGGATGACGTCAG
TCATCATGCTTACAACAGGCTACACATTGCTCAATGGCCATACAAGAGAGCGCACCCTCGCGGGAGCA
CGGAACCTTTAAGGCGCGGATCCGAATGATCGCACTGATCCTGAATCGAATCTGTAATCGATACAAGTA
CAGCGGAATCGTTCCGGGCGTTGTAATCAG

>166

GCGGCGGGCGGCGGACTACACGTGCAGTCGAACGGTAACAGGGAAGCAGCTTGCTGCTTCGCTGACG
AGTGGCGGACGGGTGAGTAATGTCTGGGAAACTGCCTGATGGAGGGGGATAACTACTGGAAACGGTA
GCTAATACCGCATAACGTCGCAAGACCAAAGAGGGGGACCTTCGGGCCTCTTGCCATCGGATGTGCCC
AGATGGGATTAGCTAGTAGGTGGGGTAACGGCTCACCTAGGCGACGATCCCTAGCTGGTCTGAGAGGA
TGACCAGCCACACTGGAACTGAGACACGGTCCAGACTCCTACGGGAGGCAGCAGTGGGGAATATTGC
ACAATGGGCGCAAGCCTGATGCAGCCATGCCGCGTGTATGAAGAAGGCCTTCGGGTTGTAAAGTACTT
TCAGCGGGGAGGAAGGGAGTAAAGTTAATACCTTTGCTCATTGACGTTACCCGCAGAAGAAGCACCG
GCTAACTCCGTGCCAGCAGCCGCGGTAATACGGAGGGTGCAAGCGTTAATCGGAATTACTGGGCGTAA
AGCGCACGCAGGCGGTTTGTTAAGTCAGATGTGAAATCCCCGGGCTCAACCTGGGAACTGCATCTGAT
ACTGGCAAGCTTGAGTCTCGTAGAGGGGGGTAGAATTCCAGGTGTAGCGGTGAAATGCGTAGAGATCT
GGAGGAATACCGGTGGCGAAGGCGGCCCCCTGGACGAAGACTGACGCTCAGGTGCGAAAGCGTGGG
GAGCAAACAGGATTAGATACCCTGGTAGTCCACGCCGTAAACGATGTCGACTTGGAGGTTGTGCCCTT
GAGGCGTGGCTTCCGGAGCTAACGCGTTAAGTCGACCGCCTGGGGAGTACGGCCGCAAGGTTAAAAC
TCAAATGAATTGACGGGGGCCCGCACAAGCGGTGGAGCATGTGGTTTAATTCGATGCAACGCGAAGA
ACCTTACCTGGTCTTGACATCCACAGAACTTTCCAGAGATGGATATGTGCCTTCGGGAACTGTGAGAC
AGTGCTGCATGGCTGTCGTCAGCTCGTGTTGTGAAATGTTGGGTTAGTCCGCACGAGCGCACCCTATC
CTTTGTTGCCAGCGGTCCGGCCGGGACTCAAGGAGACTGCCAGTGATAACTGGAGGAAGGGGGGGAT
GACGTCAGTCATCATGACCTACGAACAGGCTACCACACGTGCTACATGGCGCATACAAAGAGAAGCG
ACCTTCGCGGAAGACACGGACTTAAGTGCGCTGTATTCCGAAGTGGAAGTCTGCACTCGACCCTGATC
CGAATCGCTAAATCGAATCGAATGTACACGCGAATATGCTTTCCCGGCGCCGTGTTATATATACGCCGCT
>114
GCGGCTGGGCGGCGGCTACACATGCAGTCGAACGGTAACAGGAAGCAGCTTGCTGCTTCGCTGACGA
GTGGCGGACGGGTGAGTAATGTCTGGGAAACTGCCTGATGGAGGGGGATAACTACTGGAAACGGTAG
CTAATACCGCATAACGTCGCAAGACCAAAGAGGGGGACCTTCGGGCCTCTTGCCATCGGATGTGCCCA
GATGGGATTAGCTAGTAGGTGGGGTAACGGCTCACCTAGGCGACGATCCCTAGCTGGTCTGAGAGGAT
GACCAGCCACACTGGAACTGAGACACGGTCCAGACTCCTACGGGAGGCAGCAGTGGGGAATATTGCA
CAATGGGCGCAAGCCTGATGCAGCCATGCCGCGTGTATGAAGAAGGCCTTCGGGTTGTAAAGTACTTT
CAGCGGGGAGGAAGGGAGTAAAGTTAATACCTTTGCTCATTGACGTTACCCGCAGAAGAAGCACCGG
CTAACTCCGTGCCAGCAGCCGCGGTAATACGGAGGGTGCAAGCGTTAATCGGAATTACTGGGCGTAAA
GCGCACGCAGGCGGTTTGTTAAGTCAGATGTGAAATCCCCGGGCTCAACCTGGGAACTGCATCTGATA
CTGGCAAGCTTGAGTCTCGTAGAGGGGGGTAGAATTCCAGGTGTAGCGGTGAAATGCGTAGAGATCTG
GAGGAATACCGGTGGCGAAGGCGGCCCCCTGGACGAAGACTGACGCTCAGGTGCGAAAGCGTGGGG
AGCAAACAGGATTAGATACCCTGGTAGTCCACGCCGTAAACGATGTCGACTTGGAGGTTGTGCCCTTG
AGGCGTGGCTTCCGGAGCTAACGCGTTAAGTCGACCGCCTGGGGAGTACGGCCGCAAGGTTAAAACT
CAAATGAATTGACGGGGGCCCGCACAAGCGGTGGAGCATGTGGTTTAATTCGATGCAACGCGAAGAA
CCTTACCTGGTCTTGACATCCACAGAACTTTCCAGAGATGGATTGGTGCCTTCGGGACTGTGAGACAG
GTGCTGCATGGCTGTCGTCAGCTCGTGTGTGAAATGTTGGGTTAAGTCCCGCACGAGCGCACCCTTAT
CTTTGTTGCAGCGGTCGCGGGACTCAAGGAACTGCATGATAAACTGGAGGAAGGGTGGGGATGACG
TCAGTCATCATGCTTACGAACAGGCTACACACTGCTACATGGCCATACAAAGAGAGCGACCTCGCGAG
ACACGAACCTTAATGGCGCTGGATCCGAGTGAGTGCACCTATCTCTGATCGGAGCTCTGTAATCGTACA
AGTGACCGGGTATACTATATCCCGCCTGTTATATCACGCGCGTCA

Table S2. Comparison of the virulence genes among O157, K88, EcN, K12 and the *E. coli* 166 genomes.

Virulence factors	Related genes	*E. coli* O157:H7 str. EC4115 (EHEC)	*E. coli* UMNK88 (ETEC)	*E. coli* str. K-12 substr. MG1655	*E. coli* Nissle 1917	*E. coli* 166
		chromosome NC_011353 (5572075 bp)	chromosome NC_017641 (5186416 bp)	chromosome NC_000913 (4639675 bp)	chromosome NZ_CP007799 (5441200 bp)	chromosome (4 376 770 bp)
Adherence						
AIDA-I type	tibA					Y
Curli fibers	cgsG					Y
	cgsE					Y
	cgsD					Y
	csgB					Y
	csgA					Y
	csgC					Y
E.coli laminin-binding fimbriae (ELF)	elfA	Y	Y	Y		Y
	elfD	Y	Y	Y		Y
	elfC		Y	Y		Y
	elfG		Y	Y		Y
EaeH	eaeH	Y	Y			
ECP	ykgK/ecpR	Y	Y	Y	Y	
	yagZ/ecpA	Y	Y	Y	Y	Y
	yagY/ecpB	Y	Y	Y	Y	Y
	yagX/ecpC	Y	Y	Y	Y	Y
	yagW/ecpD	Y	Y	Y	Y	Y
	yagV/ecpE	Y	Y	Y	Y	
EhaA, AIDA-I type	ehaA	Y				
EhaB, AIDA-I type	ehaB		Y			
F1C fimbriae	focA				Y	Y
	focG				Y	
	focH				Y	
Hemorrhagic E.coli pilus (HCP)	hcpA	Y	Y	Y		
	hcpB	Y	Y	Y		
	hcpC	Y	Y	Y		
P fimbriae	papX				Y	
	papD					Y
	papC					Y
	papB				Y	
	papI				Y	
Paa	paa	Y				
S fimbriae	sfaC					Y
	sfaD					Y
	sfaF					Y
	sfaG					Y
Type 1 fimbriae	fimB	Y		Y	Y	
	fimE	Y		Y	Y	
	fimA	Y		Y	Y	
	fimI	Y		Y	Y	
	fimC	Y		Y	Y	Y
	fimD	Y		Y	Y	Y
	fimF	Y		Y	Y	Y
	fimG	Y		Y	Y	Y
	fimH	Y		Y	Y	Y
UpaG adhesin, trimeric AT	upaG/ehaG	Y				
Invasion						
Ibes	ibeB	Y	Y			
	ibeC	Y	Y			
Effector delivery system						
ACE T6SS	aec32		Y			
	aec31	Y	Y			
	aec30		Y			
	aec29	Y	Y			
	aec28	Y	Y			
	aec27/clpV	Y	Y			
	aec26	Y	Y			
	aec25	Y	Y			
	aec24	Y	Y			
	aec23	Y	Y			
	aec22	Y	Y			
	-	Y	Y			
	aec19	Y	Y			
	aec18		Y			
	aec17	Y	Y			
	aec16	Y	Y			
	aec15	Y	Y			
Intimin	eae	Y				
TTSS	escF	Y				
	cesD2	Y				
	espB	Y				
	espD	Y				
	espA	Y				
	sepL	Y				
	escD	Y				
	cesT	Y				
	cesF	Y				
	sepQ/escQ	Y				
	escO	Y				
	escN	Y				
	escV	Y				
	cesL	Y				
	escJ	Y				
	sepD	Y				
	escC	Y				
	glrR	Y			Y	Y
	etgA	Y				
	escU	Y				
	escT	Y				
	escS	Y				
	escR	Y				
	escL	Y				
	escK	Y				
	cesAB	Y				
	escE	Y				
	rorf1	Y				
TTSS secreted effectors	espB	Y				
	espG	Y				
	espH	Y				
	espF	Y				
	tir	Y				
	map	Y			Y	Y
	espFu/tccP	Y				
	espJ	Y				
	nleA/espI	Y				
	nleC	Y				
	espK	Y				
	espL1	Y				
	espL2	Y				
	espL4	Y				
	espM1	Y				
	espN	Y				
	espR1	Y				
	espR3	Y				
	espR4	Y				
	espW	Y				
	espX1	Y				
	espX2	Y				
	espX4	Y				
	espX5	Y				
	espX6	Y				
	espX7/nleL	Y				
	espY2	Y				
	espY3	Y				
	espY4	Y				
	nleB1	Y				
	nleB2	Y				
	nleE	Y				
	nleG-1	Y				
	nleG2-3	Y				
	nleG2-4	Y				
	nleG-3	Y				
	nleG5-1	Y				
	nleG5-2	Y				
	nleG6-2	Y				
	nleG6-3	Y				
	nleG7	Y				
	nleH1	Y				
	nleH2	Y				
Vacuolating autotransporter gene Vat	vat				Y	
Exotoxin						
α-Hemolysin	hlyD				Y	
Hemolysin/cytolysin A	hlyE/clyA	Y			Y	Y
Stx	stx2A	Y				
	stx2B	Y				
Biofilm						
Antigen 43, AIDA-I type	agn43			Y	Y	
Cah, AIDA-I type	cah	Y	Y		Y	
Nutritional/Metabolic factor						
Aerobactin	iutA				Y	
Chu	chuS	Y			Y	
	chuA	Y			Y	
	chuT	Y			Y	
	chuW	Y				
	chuX	Y				
	chuY	Y			Y	
	chuU	Y				
Iron/manganese transport	sitA				Y	
	sitB				Y	
	sitC				Y	
	sitD				Y	
Salmochelin siderophore	iroN				Y	
	iroE				Y	
	iroD				Y	
	iroC				Y	
	iroB				Y	
Yersiniabactin siderophore	ybtS				Y	
	ybtX				Y	
	ybtQ				Y	
	ybtP				Y	
	ybtA				Y	
	irp2				Y	
	irp1				Y	
	ybtU				Y	
	ybtT				Y	
	ybtE				Y	
	fyuA				Y	
Ler	ler	Y				
Others						

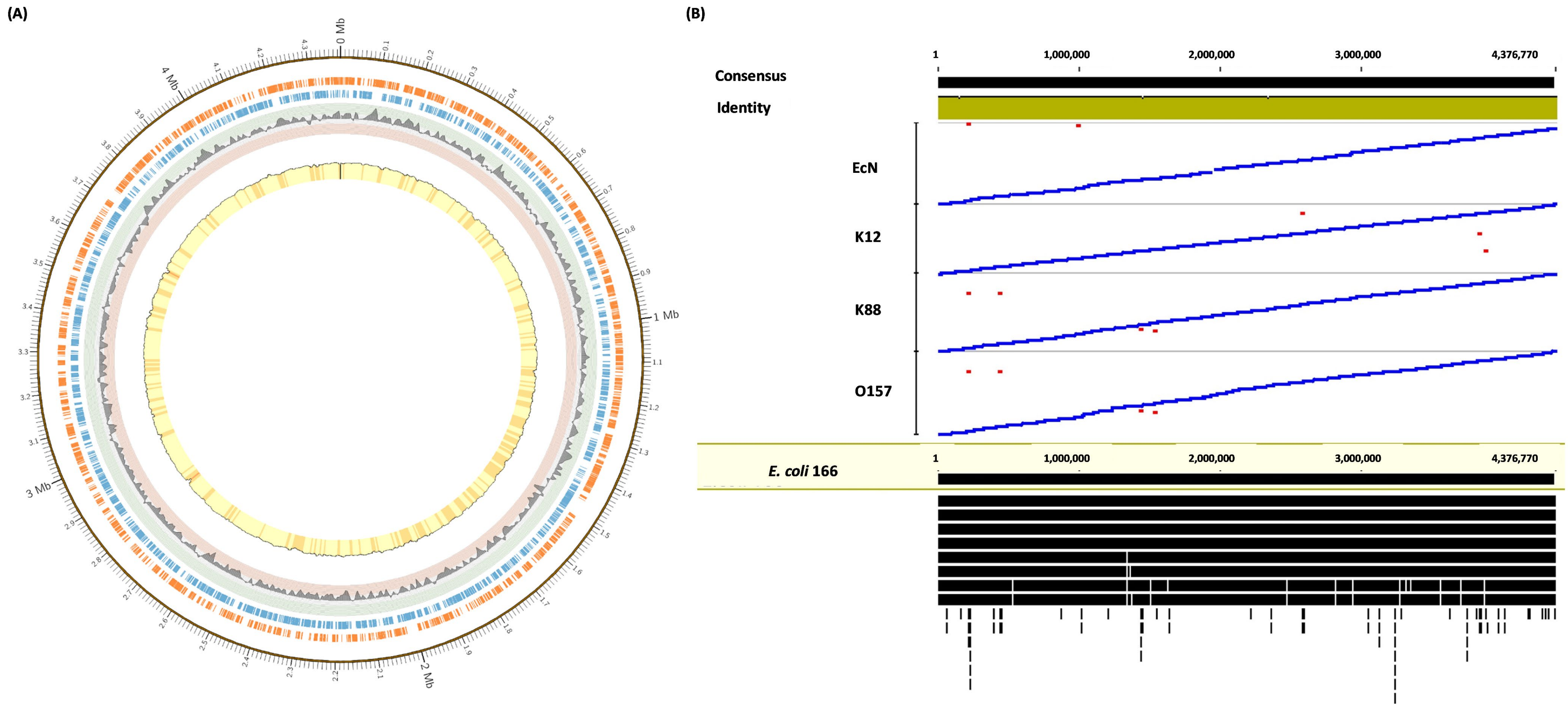

Figure S1. Genome circle of *E. coli* 166 and LASTZ dot plots comparison. (A) The concentric circles show from outside to inside: 1st circle, Genomic sequence information. 2nd circle, Coding sequences (CDS) and non-coding RNA regions (rRNA, tRNA) from the reference genome. The outer layer represents the positive strand, and the inner layer represents the negative strand. 3rd circle, GC skew curve with a 2,000 bp sliding window. The dashed line represents GC skew = 0. 4th circle, GC content curve with a 2,000 bp sliding window. The dashed line represents the average GC content of the reference genome. **(B)** LASTZ (Large-Scale Genome Alignment Tool) analysis compare the consensus in genomes among *E. coli* 166 and other four *E. coli* reference strains.
